# Slow wave activity across sleep-night could predict levodopa-induced dyskinesia

**DOI:** 10.1038/s41598-023-42604-1

**Published:** 2023-09-19

**Authors:** Ninfa Amato, Serena Caverzasio, Mauro Manconi, Claudio Staedler, Alain Kaelin-Lang, Salvatore Galati

**Affiliations:** 1 Parkinson Disease and Movement Disorder Center, Neurocenter of Southern Switzerland, EOC, Via Tesserete 46, 6903 Lugano, Switzerland; 2 Faculty of Biomedical Sciences, Università Della Svizzera Italiana (USI), Via Giuseppe Buffi 13, Lugano, Switzerland; 3 Department of Neurology, Inselspital, Bern University Hospital, Freiburgstrasse 18, Bern, Switzerland

**Keywords:** Non-REM sleep, Parkinson's disease

## Abstract

A disruption in the slow wave activity (SWA) mediated synaptic downscaling process features Parkinson's disease (PD) patients presenting levodopa-induced dyskinesia (LID). To corroborate the role of SWA in LID development, 15 PD patients with LID, who underwent a polysomnography before LID’s appearance, were included. Slow wave sleep epochs were extracted, combined and segmented into early and late sleep. SWA power was calculated. A linear regression model established that the SWA overnight decrease could predict the time to the emergence of LID. Our finding supports the link between SWA-mediated synaptic downscaling and the development of LID. If confirmed, it could pave the way to the study of possible sleep targeted therapies able to protect PD patients from LID development.

## Introduction

Levodopa-induced dyskinesia (LID) is the major long term therapy related complication in Parkinson’s disease (PD), characterized by involuntary hyperkinetic movements, including chorea, dystonia, and athetosis. LID can be functionally explained as intruding motor programs in selected neuronal response operated by the basal ganglia, leading to a perturbation of the desired motor planning by enduring signal^1^. At present, no clinically effective pharmacological therapies are able to alleviate LID without worsening parkinsonism. An alteration of synaptic plasticity has been proposed to be at the root of LID development^2^.

During sleep, neurons have to normalize total synaptic strength, thus the daytime synaptic upscaling is followed by a selective downscaling process. The amount of SWA during slow wave sleep (SWS-SWA), which is mainly produced in the first part of the night and gradually decreases throughout the night, represents the main electrophysiological marker, as well as a contributor, of this homeostatic process, which in turn is strengthen by the amount of wakefulness preceding the sleep episode^3^. Although sleep disruption is widely reported in PD ^4^, it’s still unclear how sleep itself impacts the disease course and, overall, how sleep manipulation could be helpful in ameliorating PD symptoms and neurodegeneration.

Previous finding suggested a link between SWA alteration and LID development: a reduced amount of SWA has been observed in both, rodent models and persons with PD^5^, especially in those affected by LID, in whom the physiological SWA-mediated downscaling process is impaired^6^.

We hypothesize the existence of a causal relationship between SWA alteration and LID development, with an impairment in the SWA-mediated downscaling process favoring the development of dyskinesia. Aim of this study is to assess the possible role of SWS-SWA as a marker of LID development in PD patients.

## Results

**Figure 1:**
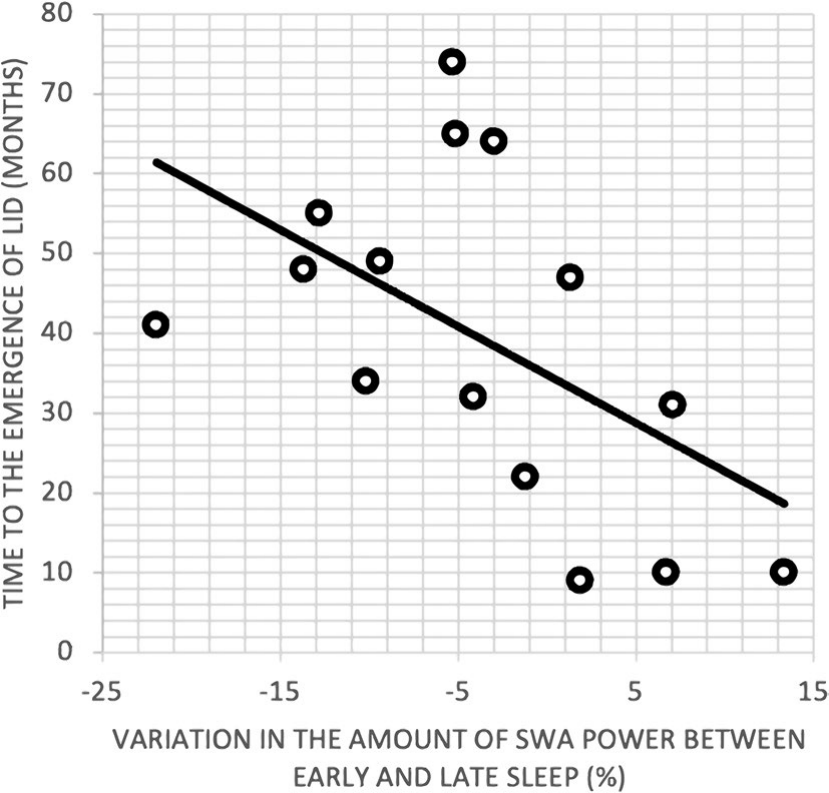
*Correlation* – Relationship between slow wave activity (SWA) overnight decrease and time to the emergence of Levodopa-induced dyskinesia (TLID). SWA overnight decrease expressed in percentage on the x-axis (negative values indicate a greater SWA power overnight reduction). TLID expressed in months on the y-axis.

We observed a significant correlation (Fig. 1) between the overnight SWS-SWA power decrease and the time to the emergence of LID (r = − 0.533, *p* = 0.04).

No significant correlations have been observed between TLID and DD, between TLID and LEDD at PSG nor between LEDD at the time of the last therapy adjustment (LTA) before LID appearance and time to the emergence of LID since that moment (TLID at LTA).

The linear regression model established that the overnight SWS-SWA power decrease over central regions could be able to predict the time to the emergence of LID (*p* = 0.04).

## Discussion

A reduced amount of SWS has been consistently reported in Parkinsonian animal models and patients affected by PD^4, 5^. Aim of this retrospective study was to assess the possible role of SWS-SWA as a marker of LID development in PD patients.

We found an association between SWS-SWA alterations and time to the emergence of LID in our cohort of PD patients. This finding is in line with previous evidence, showing that PD patients with higher content of SWS-SWA present a slower motor progression of their axial symptoms^7^.

Recent evidence^8^ demonstrated that LID is most likely to be associated with the degree of neurodegeneration than the duration of levodopa treatment. Interestingly, herein, we didn’t find any association between LEDD at the PSG and time to the emergence of LID from the moment of the recording, nor between LEDD at the last therapy adjustment before LID and time to the emergence of LID from that moment. It's important to note that all the patients in our study were on stable therapy when LID appeared (see Supplementary Table S1 online).

We speculate that the nature of the association between SWS alterations, observed in PD patients with LID, and the appearance of dyskinesia, could be causative or at least facilitative. Specifically, this association may rely on the dual role of SWS: on one side, a reduction in the SWS-SWA amount in the first part of the sleep could impair the sleep related synaptic downscaling process leading to an aberrant plasticity and then to the LID development; on the other side, the decreased amount of SWS could compromise the cleaning of neurotoxic protein aggregates^9, 10^, mainly occurring during SWS via the glymphatic system, and may thus result in an acceleration of the neurodegenerative process.

Indeed, a growing number of studies pointed out the role of SWS in avoiding the accumulation of beta-amyloid or alpha-synuclein associated with neurodegenerative disease, such as Alzheimer’s disease and PD, through glymphatic clearance^9, 10^.

We recently observed that, regardless the severity of the disease, PD patients with LID have alteration in the downscaling process, with a low SWS-SWA amount in the first part of the night, which remains stable across the night. Herein, we extended our previous observation demonstrating that the overnight reduction in the SWS-SWA power could be able to predict the appearance of LID. The lack of SWA-mediated plasticity normalization could represent a biomarker for LID development and could thus provide fundamental prognostic information. A small sample size is a major limit of this study and further studies, with larger sample size and with proper stratification and clinical characterization, are warranted to confirm and extend our data. The clinical heterogeneity of our sample and the lack of a comparison group of patients without LID, with similar characteristics in terms of disease duration, age, gender and LEDD, represents another limit of the present study. Further studies confirming SWA alterations in LID patients and documenting no SWA dysfunction in a control group of patients with similar characteristics would be helpful in making our result stronger. Establishing a causative relationship between an abnormal downscaling process and LID could pave the way for the study of sleep targeted therapies in PD. For instance, SWA enhancement interventions could be adopted to compensate for the alteration observed in dyskinetic patients. Among the interventions reported, transcranial electric stimulation, transcranial magnetic stimulation and acoustic stimulation seem to have the highest specificity and have been already successfully applied in several populations mainly with the aim of improving cognition^12^. These techniques might be applied in dyskinetic patients to obtain an increase in the first part of the night SWA amount and the restoration of the physiological overnight SWA decrease, with the aim of delaying the appearance or reduce the severity of LID, through the re-establishment of the SWA – mediated synaptic downscaling process.

## Methods

### Subjects

**Figure 2:**
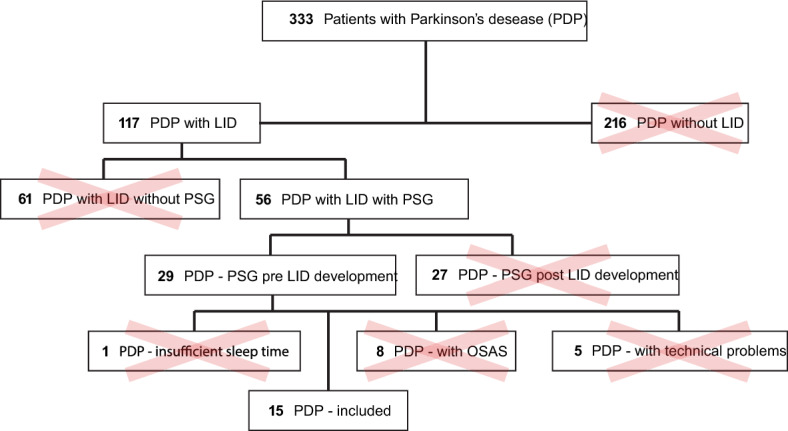
*PD patients identification process* – A total of 333 PD patients were identified, of whom 117 presented LID at the last clinical follow-up. Among PD patients with LID, 29 subjects had undergone a polysomnography (PSG) recording before its emergence. We excluded 8 patients because presenting at the PSG a moderate to severe obstructive sleep apnea syndrome (OSAS) and 1 patient because of insufficient time of sleep during the recording (< 240 min). Among the remaining 20 patients, 5 patients were excluded for technical reasons and 15 patients were included in the present study.

A retrospective screening of the local Movement Disorders Unit database was performed (Fig. 2) and 15 patients (Table 1), with a diagnosis of idiopathic PD, according to the UK PD society Brain Bank, who underwent a polysomnography (PSG) before developing LID (as detected by the examiner during clinical visits), were included. For the whole group, the time of the day with a most prominent LID was the evening. Out of the 15 patients, 11 were included in the PSG recording as volunteers for previous research projects, while the other 4 underwent the PSG due to suspicion of sleep apnea that was not confirmed. Among the 11 PSGs performed for research purposes, 8 were recorded on the second of two consecutive nights spent by patients in the sleep lab, whereas the other 3, and the 4 PSGs performed for clinical reasons, were recorded on the first and only night. To avoid any variability in sleep quality due to the first night effect, which could have had an impact on our main outcome, we split our sample in two subgroups and checked for any difference in sleep parameters. We found no differences between the two subgroups, so we collapsed them back in one group and considered the whole group for the analysis. The study was approved by the Local Ethics Committee (Comitato Etico Cantonale Ticino) and conducted in compliance with the current version of the Declaration of Helsinki, the ICH-GCP and all national regulatory requirements. Patients were informed and gave their consent to the use of their data.

**Table 1:** PD patients included: demographic and clinical characteristics (*DD*, disease duration; *TLID,* time to the emergence of LID; m, months; *TST,* total sleep time; *SL,* sleep latency; *SE,* sleep efficiency).

ID	Age	Sex	DD at PSG (m)	TLID at PSG (m)	LEDD at PSG (m)	TST	SL	SE	N1	N2	N3	REM	MDS-UPDRS III	H&Y
001	54	M	53	49	280	355	0.4	72.6	46.5	183	63	62.5	15	1.5
002	60	M	9	34	300	289	3.1	58.8	53.5	165	25	45.5	19	1.5
003	65	F	12	22	0	370	13	84.5	27.5	215	51	77	14	2
004	71	M	24	65	450	376	40.6	83	5	334	18.5	18	12	1.5
005	75	F	104	9	550	237	27	61.3	39.5	115	62	20.5	25	2
006	71	F	99	32	739	359	28.6	80.3	1	181	104	57.4	24	2
007	80	M	24	48	300	364	11.6	87.4	20	205	69.6	35.9	7	1
008	83	F	28	64	300	230	110	43.5	7	143	54	26	43	2.5
009	82	M	85	10	800	285	34	63.6	17.1	109	73	84.9	7	1
010	74	M	65	10	210	185	21.3	47	20.5	54.5	71.5	38	31	2
011	75	F	90	41	600	327	13.2	65.5	39	203	0	84.5	10	1.5
012	81	F	196	47	150	481	2.2	97.1	31.4	232	215	3.5	20	2
013	57	M	23	74	100	338	21.1	76.7	72.5	128	48	90	24	2
014	66	M	37	55	300	286	1.1	61.3	40.6	97.5	114	33.5	15	1
015	73	F	117	31	700	350	6.1	86.5	71.8	238	12.6	27.7	12	2

### EEG-PSG recordings

Patients underwent a nocturnal PSG, recorded in a standard sound-attenuated sleep laboratory (noise level to a maximum of 30 dB). All the recordings included scalp EEG channels (19 to 256), submental electromyogram, electro-oculogram, electrocardiogram, cardiorespiratory channels, and electromyogram of the right and left tibialis anterior. Sleep scoring was performed according to standardized criteria (AASM).

### EEG-PSG analysis

NREM sleep EEG data were extracted, rejecting epochs containing arousals, combined and subdivided in 2 equal parts, early and late sleep respectively, which were further analyzed as described in^5^. The overnight SWS-SWA power decrease, reflecting the synaptic downscaling process, was calculated as the percentage change: {[(late_sleep-early_sleep)/early_sleep]*100}. The relationship between time to the emergence of LID (TLID) and SWS-SWA power decrease over central regions (channels C3-C4), was then investigated.

### Statistical analysis

Before building a linear regression model, the variables’ behavior was graphically investigated by means of box plots in order to spot any possible outlier observations in the variables, which could affect the direction as well as the slope of the line of best fit. Then, a Pearson correlation was performed to investigate the level of the linear dependence between the amount of the overnight SWS-SWA decrease and the response variable, between disease duration (DD) and TLID, between the amount of levodopa intake (levodopa equivalent daily dose – LEDD) and TLID, and between the amount of LEDD at the last therapy adjustment (LTA) before LID emergency and the time to the emergence of LID at LTA.

Then, TLID, expressed in months, was considered as the dependent variable (response), while the amount of the overnight SWS-SWA power decrease was considered as the independent variable (predictor). A linear regression was run in order to assess if the values of the response variable could be predicted based on the values of the predictor variable. Normality of the residuals was assessed using a QQ-plot. Homoscedasticity of the residuals was checked by means of the Breush-Pagan test. Results were considered significant at a level of p < 0.05. Statistical analysis was performed using R^11^.

### Supplementary Information

Supplementary Table S1.

## Data Availability

The datasets are available from the corresponding author on reasonable request.
