# Supplementary material for: Slow wave activity across sleep-night could predict levodopa-induced dyskinesia
Source: Sci Rep. 2023 Sep 19;13:15468. doi: 10.1038/s41598-023-42604-1 (PMC10509191; doi:10.1038/s41598-023-42604-1)
Supplement: Supplementary file 1 — Supplementary Table S1. [file 41598_2023_42604_MOESM1_ESM.docx]

| **ID** | **Time to LID** | **Madopar®**  **cpr** | **Madopar®**  **liq** | **Madopar®**  **DR** | **Azilect®** | **Neupro®** | **Sinemet®** | **Sinemet®**  **CR** | **Sifrol®** | **Sifrol®**  **ER** | **Xadago®** | **Requip®** | **Stalevo®** | **LEDD** |
| --- | --- | --- | --- | --- | --- | --- | --- | --- | --- | --- | --- | --- | --- | --- |
|  | (months) | levodopa, benserazide (4:1) | levodopa, benserazide (4:1) | levodopa, benserazide (4:1) | rasagiline | rigotine | carbidopa,  levodopa  (1:4) | carbidopa, levodopa  (1:4) | pramipexole | pramipexole | safinamide | ropinirole | levodopa,  carbidopa (1:4) entacapone (*200)* |  |
| **001** |  |  |  |  |  |  |  |  |  |  |  |  |  |  |
| (PSG) | 49 |  |  |  | 1 mg  1/day  (1) | 6 mg  1/day  (1) |  |  |  |  |  |  |  | 280 |
|  | 40 |  |  |  | 1 mg  1/day  (1) | 6 mg  1/day  (1) |  |  |  |  |  |  |  | 280 |
|  | 37 |  |  |  | 1 mg  1/day  (1) | 8 mg  1/day  (1) |  |  |  |  |  |  |  | 340 |
|  | 31 |  |  |  | 1 mg  1/day  (1) | 8+4 mg  1/day  (1) |  |  |  |  |  |  |  | 460 |
|  | 28 |  |  |  | 1 mg  1/day  (1) | 8+4 mg  1/day  (1) |  |  |  |  |  |  |  | 460 |
|  | 23 |  |  |  | 1 mg  1/day  (1) | 8+4 mg  1/day  (1) |  |  |  |  |  |  |  | 460 |
|  | 20 |  |  |  | 1 mg  1/day  (1) | 8+4 mg  1/day  (1) | 25/100 mg  3/day  (1 - 1 - 1) |  |  |  |  |  |  | 760 |
|  | 14 |  |  |  | 1 mg  1/day  (1) | 8+4 mg  1/day  (1) | 25/100 mg  3/day  (1 - 1 - 1) |  |  |  |  |  |  | 760 |
|  | 8 |  |  |  | 1 mg  1/day  (1) | 8+4 mg  1/day  (1) | 25/100 mg  3/day  (1 - 1 - 1) |  |  |  |  |  |  | 760 |
|  | 3 |  |  |  | 1 mg  1/day  (1) | 8+4 mg  1/day  (1) | 25/100 mg  3/day  (1 - 1 - 1) |  |  |  |  |  |  | 760 |
| (LID) | 0 |  |  |  | 1 mg  1/day  (1) | 8+4 mg  1/day  (1) | 25/100 mg  3/day  (1 - 1 - 1) |  |  |  |  |  |  | 760 |
|  |  |  |  |  |  |  |  |  |  |  |  |  |  |  |
| **002** |  |  |  |  |  |  |  |  |  |  |  |  |  |  |
| (PSG) | 34 | 250 mg  3/day  (1/2 - 1/2 - 1/2) |  |  |  |  |  |  |  |  |  |  |  | 300 |
|  | 31 | 250 mg  3/day  (1/2 - 1/2 - 1/2) |  |  |  |  |  |  |  |  |  |  |  | 300 |
|  | 26 | 250 mg  3/day  (1/2 - 1/2 - 1/2) |  |  |  |  |  |  |  | 0.75 mg  1/day  (1) |  |  |  | 375 |
|  | 22 | 250 mg  3/day  (1/2 - 1/2 - 1/2) |  |  |  |  |  |  |  | 0.75 mg  1/day  (1) |  |  |  | 375 |
|  | 19 | 250 mg  3/day  (1/2 - 1/2 - 1/2) |  |  |  |  |  |  |  | 0.75 mg  1/day  (1) |  |  |  | 450 |
|  | 16 | 250 mg  3/day  (1/2 - 1/2 - 1/2) |  |  |  |  |  |  |  | 0.75 mg  1/day  (1) |  |  |  | 450 |
|  | 12 | 250 mg  3/day  (3/4 - 3/4 - 3/4) |  |  |  |  |  |  |  | 0.75 mg  1/day  (1) |  |  |  | 450 |
|  | 10 | 250 mg  3/day  (3/4 - 3/4 - 3/4) |  |  |  |  |  |  |  | 0.75 mg  1/day  (1) |  |  |  | 450 |
|  | 7 | 250 mg  3/day  (3/4 - 3/4 - 3/4) |  |  |  |  |  |  |  | 0.75 mg  1/day  (1) |  |  |  | 450 |
|  | 4 | 250 mg  4/day  (3/4 - 1/2 - 3/4 - 1/2) |  |  |  |  |  |  |  |  |  |  |  | 500 |
| (LID) | 0 | 250 mg  4/day  (3/4 - 1/2 - 3/4 - 1/2) |  |  |  |  |  |  |  |  |  |  |  | 500 |
|  |  |  |  |  |  |  |  |  |  |  |  |  |  |  |
| **003** |  |  |  |  |  |  |  |  |  |  |  |  |  |  |
| (PSG) | 22 |  |  |  |  |  |  |  |  |  |  |  |  | 0 |
|  | 19 |  |  |  |  |  |  |  |  |  |  |  |  | 0 |
|  | 12 |  |  |  |  |  |  |  |  |  |  |  |  | 0 |
|  | 10 |  |  |  |  |  |  |  |  |  |  |  |  | 0 |
|  | 9 | 125 mg  3/day  (1/2 - 1/2 - 1/2) |  |  |  |  |  |  |  |  |  |  |  | 150 |
|  | 5 | 125 mg  3/day  (1/2 - 1/2 - 1/2) |  |  |  |  |  |  |  |  |  |  |  | 150 |
|  | 1 | 125 mg  3/day  (1/2 - 1/2 - 1/2) |  |  |  |  |  |  |  |  |  |  |  | 150 |
| (LID) | 0 | 125 mg  3/day  (1/2 - 1/2 - 1/2) |  |  |  |  |  |  |  |  |  |  |  | 150 |
|  |  |  |  |  |  |  |  |  |  |  |  |  |  |  |
| **004** |  |  |  |  |  |  |  |  |  |  |  |  |  |  |
| (PSG) | 65 | 125 mg  3/day  (1.5 - 1.5 - 1.5) |  |  |  |  |  |  |  |  |  |  |  | 450 |
|  | 60 | 125 mg  3/day  (1 - 1 - 1) |  |  |  |  |  |  |  |  |  |  |  | 300 |
|  | 56 | 125 mg  3/day  (1 - 1 - 1) |  |  |  |  |  |  |  |  |  |  |  | 300 |
|  | 50 | 125 mg  3/day  (1 - 1 - 1) |  |  |  |  |  |  |  |  |  |  |  | 300 |
|  | 46 | 125 mg  3/day  (1 - 1 - 1) |  |  |  |  |  |  |  |  |  |  |  | 300 |
|  | 40 | 125 mg  3/day  (1 - 1 - 1) |  |  |  |  |  |  |  |  |  |  |  | 300 |
|  | 34 | 125 mg  3/day  (1 - 1 - 1) |  |  |  |  |  |  |  |  |  |  |  | 300 |
|  | 32 | 125 mg  3/day  (1 - 1 - 1) |  |  |  | 4 mg  1/day  (1) |  |  |  |  |  |  |  | 420 |
|  | 24 | 125 mg  3/day  (1 - 1 - 1) |  |  |  | 4 mg  1/day  (1) |  |  |  |  |  |  |  | 420 |
|  | 20 | 125 mg  3/day  (1 - 1 - 1) |  |  |  | 4 mg  1/day  (1) |  |  |  |  |  |  |  | 420 |
|  | 17 | 125 mg  3/day  (1 - 1 - 1) |  |  |  | 4 mg  1/day  (1) |  |  |  |  |  |  |  | 420 |
|  | 11 | 125 mg  4/day  (1 - 1 - 1 - 0.5) |  |  |  | 4 mg  1/day  (1) |  |  |  |  |  |  |  | 470 |
|  | 9 |  |  |  |  | 6 mg  1/day  (1) |  |  |  |  |  |  | 100/25/200 mg  4/day  (1 - 1 - 1 - 1) | 712 |
|  | 8 |  |  |  |  | 6 mg  1/day  (1) |  |  |  |  |  |  | 100/25/200 mg  4/day  (1 - 1 - 1 - 1) | 712 |
|  | 4 |  |  |  |  | 6 mg  1/day  (1) |  |  |  |  |  |  | 100/25/200 mg  4/day  (1 - 1 - 1 - 1) | 712 |
| (LID) | 0 |  |  |  |  | 6 mg  1/day  (1) |  |  |  |  |  |  | 100/25/200 mg  4/day  (1 - 1 - 1 - 1) | 712 |
|  |  |  |  |  |  |  |  |  |  |  |  |  |  |  |
| **005** |  |  |  |  |  |  |  |  |  |  |  |  |  |  |
| (PSG) | 9 | 125 mg  2/day  (0.5 - 0.5) |  |  |  |  |  |  |  | 3 mg  1/day  + 1.5 mg 1/day  (1-1) |  |  |  | 550 |
|  | 8 | 125 mg  2/day  (0.5 - 0.5) |  |  |  |  |  |  |  | 3 mg  1/day  + 1.5 mg  1/day  (1-1) |  |  |  | 550 |
|  | 6 | 125 mg  4/day  (2 - 2 - 2 - 1.5) |  |  |  |  |  | 25/100 m  1/day  (1) |  | 3 mg  1/day  (1) |  |  |  | 1125 |
|  | 4 | 125 mg  4/day  (2 - 2 - 2 - 1.5) |  |  |  |  |  | 25/100 mg  1/day  (1) |  | 3 mg  1/day  (1) |  |  |  | 1125 |
| (LID) | 0 | 125 mg  4/day  (2 - 2 - 2 - 1.5) |  |  |  |  |  | 25/100 mg  1/day  (1) |  | 3 mg  1/day  (1) |  |  |  | 1125 |
|  |  |  |  |  |  |  |  |  |  |  |  |  |  |  |
| **006** |  |  |  |  |  |  |  |  |  |  |  |  |  |  |
| (PSG) | 32 |  |  |  |  | 3 mg  1/day  (1) |  | 50/200 mg  1/day  (1) |  |  |  |  | 125/31.25/200 mg  3/day  (1 - 1 - 1) | 739 |
|  | 28 |  |  |  |  | 3 mg  1/day  (1) |  | 50/200 mg  1.5/day  (1) |  |  |  |  | 125/31.25/200 mg  3/day  (1 - 1 - 1) | 814 |
|  | 25 |  |  |  |  | 3 mg  1/day  (1) |  | 50/200 mg  1/day  (1) |  |  |  |  | 125/31.25/200 mg  4/day  (1 - 1 - 1 - 1) | 905 |
|  | 21 |  |  |  |  | 3 mg  1/day  (1) |  | 50/200 mg  1/day  (1) |  |  |  |  | 125/31.25/200 mg  4/day  (1 - 1 - 1 - 1) | 905 |
|  | 18 |  |  |  |  | 3 mg  1/day  (1) |  | 50/200 mg  1/day  (1) |  |  |  |  | 125/31.25/200 mg  4/day  (1 - 1 - 1 - 1) | 905 |
|  | 15 |  |  |  |  |  |  | 50/200 mg  1/day  (1) |  |  | 50 mg  1/day  (1) |  | 125/31.25/200 mg  4/day  (1 - 1 - 1 - 1) | 915 |
|  | 12 |  |  |  |  |  |  | 50/200 mg  1/day  (1) |  |  | 50 mg  1/day  (1) |  | 125/31.25/200 mg  4/day  (1 - 1 - 1 - 1) | 915 |
|  | 10 |  |  |  |  |  |  | 50/200 mg  1/day  (1) |  |  | 100 mg  1/day  (1) |  | 125/31.25/200 mg  4/day  (1 - 1 - 1 - 1) | 915 |
|  | 9 |  |  |  |  |  |  | 50/200 mg  1/day  (1) |  |  | 100 mg  1/day  (1) |  | 125/31.25/200 mg  4/day  (1 - 1 - 1 - 1) | 915 |
|  | 5 |  |  |  |  |  |  | 50/200 mg  1/day  (1) |  |  | 100 mg  1/day  (1) |  | 125/31.25/200 mg  4/day  (1 - 1 - 1 - 1) | 915 |
|  | 2 |  |  |  |  |  |  | 50/200 mg  1/day  (1) |  |  | 100 mg  1/day  (1) |  | 125/31.25/200 mg 4/day  (1 - 1 - 1 - 1) | 915 |
| (LID) | 0 |  |  |  |  |  |  | 50/200 mg  1/day  (1) |  |  | 100 mg  1/day  (1) |  | 125/31.25/200 mg  4/day  (1 - 1 - 1 - 1) | 915 |
|  |  |  |  |  |  |  |  |  |  |  |  |  |  |  |
| **007** |  |  |  |  |  |  |  |  |  |  |  |  |  |  |
| (PSG) | 48 | 125 mg  3/day  (1 - 1 - 1) |  |  |  |  |  |  |  |  |  |  |  | 300 |
|  | 38 | 125 mg  3/day  (1 - 1 - 1) | 62.5 mg  1/day  (1) |  |  |  |  |  |  |  |  |  |  | 350 |
|  | 35 | 125 mg  3/day  (1.5 - 1 - 1.5) | 62.5 mg  1/day  (1) |  |  |  |  |  |  |  | 100 mg  1/day  (1) |  |  | 550 |
|  | 31 | 125 mg  3/day  (1.5 - 1 - 1) | 62.5 mg  0.5/day  (1) |  |  |  |  |  |  |  | 100 mg  1/day  (1) |  |  | 475 |
|  | 27 | 125 mg  3/day  (1.5 - 1 - 1) | 62.5 mg  0.5/day  (1) |  |  |  |  | 25/100 mg  1/day  (1) |  |  | 100 mg  1/day  (1) |  |  | 550 |
|  | 26 | 125 mg  3/day  (1.5 - 1 - 1) | 62.5 mg  0.5/day  (1) |  |  |  |  | 25/100 mg  1/day  (1) |  |  | 100 mg  1/day  (1) |  |  | 550 |
|  | 21 | 125 mg  3/day  (1.5 - 1 - 1) | 62.5 mg  0.5/day  (1) |  |  |  |  | 25/100 mg  1/day  (1) |  |  | 100 mg  1/day  (1) |  |  | 550 |
|  | 14 | 125 mg  3/day  (1.5 - 1 - 1) | 62.5 mg  0.5/day  (1) |  |  |  |  | 25/100 mg  1/day  (1) |  |  | 100 mg  1/day  (1) |  |  | 550 |
|  | 9 | 125 mg  4/day  (1 - 1 - 1 - 1) | 62.5 mg  0.5/day  (1) |  |  |  |  | 25/100 mg  1/day  (1) |  |  | 100 mg  1/day  (1) |  |  | 600 |
|  | 6 | 125 mg  4/day  (1 - 1 - 1 - 1) | 62.5 mg  1/day  (1) |  |  | 4 mg  1/day  (1) |  | 25/100 mg  1/day  (1) |  |  | 100 mg  1/day  (1) |  |  | 745 |
|  | 4 | 125 mg  4/day  (1 - 1 - 1 - 1) | 62.5 mg  1/day  (1) |  |  | 4 mg  1/day  (1) |  | 25/100 mg  1/day  (1) |  |  | 100 mg  1/day  (1) |  |  | 745 |
|  | 2 | 125 mg  4/day  (1 - 1 - 1 - 1) | 62.5 mg  1/day  (1) |  |  | 4 mg  1/day  (1) |  | 25/100 mg  1/day  (1) |  |  | 100 mg  1/day  (1) |  |  | 745 |
| (LID) | 0 | 125 mg  4/day  (1 - 1 - 1 - 1) | 125 mg  1/day  (1) |  |  | 4 mg  1/day  (1) |  | 25/100 mg  1/day  (1) |  |  | 100 mg  1/day  (1) |  |  | 795 |
|  |  |  |  |  |  |  |  |  |  |  |  |  |  |  |
| **008** |  |  |  |  |  |  |  |  |  |  |  |  |  |  |
| (PSG) | 64 |  |  |  |  |  | 25/100 mg  3/day  (1 - 1 - 1) |  |  |  |  |  |  | 300 |
|  | 63 |  |  |  |  |  | 25/100 mg  3/day  (1 - 1 - 1) |  |  |  |  |  |  | 300 |
|  | 62 |  |  |  |  |  | 25/100 mg  4/day  (1 - 1 - 1 - 1) |  | 0.125 mg  1/day  (1) |  |  |  |  | 413 |
|  | 61 |  |  |  |  |  | 25/100 mg  4/day  (1 - 1 - 1 - 1) |  | 0.125 mg  1/day  (1) |  |  |  |  | 413 |
|  | 58 |  |  |  |  |  | 25/100 mg  4/day  (1 - 1 - 1 - 1) |  | 0.125 mg  1/day  (1) |  |  |  |  | 413 |
|  | 57 |  |  |  |  |  | 25/100 mg  4/day  (1 - 1 - 1 - 1) |  | 0.125 mg  1/day  (1) |  |  |  |  | 413 |
|  | 53 |  |  |  |  |  | 25/100 mg  4/day  (1 - 1 - 1 - 1) |  | 0.125 mg  1/day  (1) |  |  |  |  | 413 |
|  | 48 |  |  |  |  |  | 25/100 mg  4/day  (1 - 1 - 1 - 1) |  | 0.125 mg  1/day  (1) |  |  |  |  | 413 |
|  | 44 |  |  |  |  |  | 25/100 mg  4/day  (1 - 1 - 1 - 1) |  | 0.125 mg  1/day  (1) |  |  |  |  | 413 |
|  | 41 |  |  |  |  | 4 mg  1/day  (1) | 25/100 mg  4/day  (1 - 1 - 1 - 1) |  |  |  |  |  |  | 520 |
|  | 39 |  |  |  |  | 4 mg  1/day  (1) | 25/100 mg  4/day  (1 - 1 - 1 - 1) |  |  |  |  |  |  | 520 |
|  | 35 |  |  |  |  | 4 mg  1/day  (1) | 25/100 mg  4/day  (1 - 1 - 1 - 1) |  |  |  |  |  |  | 520 |
|  | 32 |  |  |  |  | 4 mg  1/day  (1) | 25/100 mg  4/day  (1 - 1 - 1 - 1) |  |  |  |  |  |  | 520 |
|  | 28 |  |  |  |  |  | 25/100 mg  4/day  (1 - 1 - 1 - 1) |  |  |  |  | 4 mg  1/day  (1) |  | 520 |
|  | 26 |  |  |  |  |  | 25/100 mg  4/day  (1 - 1 - 1 - 1) |  |  |  |  | 4 mg  1/day  (1) |  | 520 |
|  | 23 |  |  |  |  |  | 25/100 mg  4/day  (1 - 1 - 1 - 1) |  |  |  |  | 4 mg  1/day  (1) |  | 520 |
|  | 20 |  |  |  |  |  | 25/100 mg  4/day  (1 - 1 - 1 - 1) |  |  |  |  | 4 mg  1/day  (1) |  | 520 |
|  | 17 |  |  |  |  |  | 25/100 mg  4/day  (1 - 1 - 1 - 1) |  |  |  |  | 4 mg  1/day  (1) |  | 520 |
|  | 13 |  |  |  |  |  | 25/100 mg  4/day  (1 - 1 - 1 - 1) |  |  |  |  | 4 mg  1/day  (1) |  | 520 |
|  | 9 |  |  |  |  |  | 25/100 mg  4/day  (1 - 1 - 1 - 1) |  |  |  |  | 4 mg  1/day  (1) |  | 520 |
|  | 5 |  |  |  |  |  | 25/100 mg  4/day  (1 - 1 - 1 - 1) |  |  |  |  | 4 mg  1/day  (1) |  | 520 |
|  | 2 |  |  |  |  |  | 25/100 mg  4/day  (1 - 1 - 1 - 1) |  |  |  |  | 4 mg  1/day  (1) |  | 520 |
| (LID) | 0 |  |  |  |  |  | 25/100 mg  4/day  (1 - 1 - 1 - 1) |  |  |  |  | 4 mg  1/day  (1) |  | 520 |
|  |  |  |  |  |  |  |  |  |  |  |  |  |  |  |
| **009** |  |  |  |  |  |  |  |  |  |  |  |  |  |  |
| (PSG) | 5 | 125 mg  8/day  (1 - 1 - 1 - 1 - 1 - 1 - 1 - 1) |  |  |  |  |  |  |  |  |  |  |  | 800 |
|  | 5 | 125 mg  8/day  (1 - 1 - 1 - 1 - 1 - 1 - 1 - 1) |  |  |  |  |  |  |  |  |  |  |  | 800 |
| (LID) | 0 | 125 mg  8/day  (1 - 1 - 1 - 1 - 1 - 1 - 1 - 1) |  |  |  |  |  |  |  |  |  |  |  | 800 |
|  |  |  |  |  |  |  |  |  |  |  |  |  |  |  |
| **010** |  |  |  |  |  |  |  |  |  |  |  |  |  |  |
| (PSG) | 10 | 62.5 mg  4/day  (1 - 1 - 1 - 1) |  |  |  |  |  |  | 0.125 mg  1/day  (1) |  |  |  |  | 210 |
|  | 6 | 62.5 mg  4/day  (1 - 1 - 1 - 1) |  |  |  |  |  |  | 0.125 mg  1/day  (1) |  |  |  |  | 210 |
|  | 2 | 62.5 mg  4/day  (1 - 1 - 1 - 1) |  |  |  |  |  |  | 0.125 mg  1/day  (1) |  |  |  |  | 210 |
| (LID) | 0 | 62.5 mg  4/day  (1 - 1 - 1 - 1) |  |  |  |  |  |  | 0.125 mg  1/day  (1) |  |  |  |  | 210 |
|  |  |  |  |  |  |  |  |  |  |  |  |  |  |  |
| **011** |  |  |  |  |  |  |  |  |  |  |  |  |  |  |
| (PSG) | 41 | 250 mg  3/day  (1 - 1 - 1) |  |  |  |  |  |  |  |  |  |  |  | 600 |
|  | 36 | 250 mg  4/day  (1 - 1 - 1 - 1) |  |  |  | 4 mg  1/day  (1) |  |  |  |  |  |  |  | 920 |
|  | 34 | 250 mg  4/day  (1 - 1 - 1 - 1) |  |  |  | 4 mg  1/day  (1) |  |  |  |  |  |  |  | 920 |
|  | 31 | 250 mg  4/day  (1 - 1 - 1 - 1) |  |  |  | 4 mg  1/day  (1) |  |  |  |  |  |  |  | 920 |
|  | 28 | 250 mg  4/day  (1 - 1 - 1 - 1) |  |  |  | 4 mg  1/day  (1) |  |  |  |  |  |  |  | 800 |
|  | 26 | 250 mg  4/day  (1 - 1 - 1 - 1) |  | 250mg  1/day  (1) |  |  |  |  |  |  |  |  |  | 950 |
|  | 23 | 250 mg  4/day  (1 - 1 - 1 - 1) |  | 250mg  1/day  (1) |  |  |  |  |  |  |  |  |  | 950 |
|  | 21 | 250 mg  4/day  (1 - 1 - 1 - 1) |  | 250mg  1/day  (1) |  |  |  |  |  |  |  |  |  | 950 |
|  | 19 | 250 mg  4/day  (1 - 1 - 1 - 1) |  | 250mg  1/day  (1) |  |  |  |  |  |  |  |  |  | 950 |
|  | 16 | 250 mg  4/day  (1 - 1 - 1 - 1) |  | 250mg  1/day  (1) |  |  |  |  |  |  |  |  |  | 950 |
|  | 13 | 250 mg  4/day  (1 - 1 - 1 - 1) |  | 250mg  1/day  (1) |  |  |  |  |  |  |  |  |  | 950 |
|  | 9 | 250 mg  4/day  (1 - 1 - 1 - 1) |  | 250mg  1/day  (1) |  |  |  |  |  |  |  |  |  | 950 |
|  | 5 | 250 mg  4/day  (1 - 1 - 1 - 1) |  | 250mg  1/day  (1) |  |  |  |  |  |  |  |  |  | 950 |
|  | 3 | 250 mg  4/day  (1 - 1 - 1 - 1) |  | 250mg  1/day  (1) |  |  |  |  |  |  |  |  |  | 950 |
| (LID) | **0** | 250 mg  4/day  (1 - 1 - 1 - 1) |  | 250mg  1/day  (1) |  |  |  |  |  |  |  |  |  | 950 |
|  |  |  |  |  |  |  |  |  |  |  |  |  |  |  |
| **012** |  |  |  |  |  |  |  |  |  |  |  |  |  |  |
| (PSG) | 47 |  |  |  |  |  |  |  |  | 1.5 mg  1/day  (1) |  |  |  | 150 |
|  | 46 |  |  |  |  |  |  |  |  | 1.5 mg  1/day  (1) |  |  |  | 150 |
|  | 43 | 125 mg  3/day  (1.5 - 1.5 - 1) |  |  |  |  |  |  |  | 1.5 mg  1/day  (1) |  |  |  | 550 |
|  | 42 | 125 mg  3/day  (1.5 - 1.5 - 1) |  |  |  |  |  |  |  | 1.5 mg  1/day  (1) |  |  |  | 550 |
|  | 39 | 125 mg  3/day  (1.5 - 1.5 - 1) |  |  |  |  |  |  |  | 1.5 mg  1/day  (1) |  |  |  | 550 |
|  | 38 | 125 mg  3/day  (1.5 - 1.5 - 1) |  |  |  |  |  |  |  | 1.5 mg  1/day  (1) |  |  |  | 550 |
|  | 36 | 125 mg  3/day  (1.5 - 1.5 - 1) |  |  |  |  |  |  |  | 1.5 mg  1/day  (1) |  |  |  | 550 |
|  | 35 | 125 mg  3/day  (1.5 - 1.5 - 1) |  |  |  |  |  |  |  | 1.5 mg  1/day  (1) |  |  |  | 550 |
|  | 31 | 125 mg  3/day  (1.5 - 1.5 - 1) |  |  |  |  |  |  |  | 1.5 mg  1/day  (1) |  |  |  | 550 |
|  | 25 | 125 mg  3/day  (1.5 - 1.5 - 1.5) |  |  |  |  |  |  |  |  |  |  |  | 450 |
|  | 24 | 125 mg  4/day  (1.5 - 1.5 - 1.5 - 1) |  |  |  |  |  |  |  |  |  |  |  | 550 |
|  | 18 | 125 mg  4/day  (1.5 - 1.5 - 1.5 - 1) |  |  |  |  |  |  |  |  |  |  |  | 550 |
|  | 12 | 125 mg  4/day  (1.5 - 1.5 - 1.5 - 1) |  |  |  |  |  |  |  |  |  |  |  | 550 |
|  | 7 | 125 mg  4/day  (1.5 - 1.5 - 1.5 - 1) |  |  |  |  |  |  |  |  |  |  |  | 550 |
|  | 4 | 125 mg  4/day  (1.5 - 1.5 - 1.5 - 1) |  |  |  |  |  |  |  |  |  |  |  | 550 |
|  | 3 | 125 mg  4/day  (1.5 - 1.5 - 1.5 - 1) |  |  |  |  |  |  |  |  |  |  |  | 550 |
| (LID) | 0 | 125 mg  4/day  (1.5 - 1.5 - 1.5 - 1) |  |  |  |  |  |  |  |  |  |  |  | 550 |
|  |  |  |  |  |  |  |  |  |  |  |  |  |  |  |
| **013** |  |  |  |  |  |  |  |  |  |  |  |  |  |  |
| (PSG) | 74 |  |  |  | 1 mg  1/day  (1) |  |  |  |  |  |  |  |  | 100 |
|  | 62 |  |  |  | 1 mg  1/day  (1) | 4 mg  1/day  (1) |  |  |  |  |  |  |  | 220 |
|  | 58 | 125 mg  3/day  (1 - 1 - 1) |  |  | 1 mg  1/day  (1) | 6 mg  1/day  (1) |  |  |  |  |  |  |  | 580 |
|  | 57 | 125 mg  3/day  (1 - 1 - 1) |  |  | 1 mg  1/day  (1) | 6 mg  1/day  (1) |  |  |  |  |  |  |  | 580 |
|  | 48 | 125 mg  3/day  (1 - 1 - 1) |  |  | 1 mg  1/day  (1) | 6 mg  1/day  (1) |  |  |  |  |  |  |  | 580 |
|  | 43 | 125 mg  4/day  (1 - 1 - 1 - 1) |  |  | 1 mg  1/day  (1) | 6 mg  1/day  (1) |  |  |  |  |  |  |  | 680 |
|  | 39 | 125 mg  4/day  (1 - 1 - 1 - 1) |  |  | 1 mg  1/day  (1) | 6 mg  1/day  (1) |  |  |  |  |  |  |  | 680 |
|  | 35 |  |  |  | 1 mg  1/day  (1) | 6 mg  1/day  (1) |  |  |  |  |  |  | 100/25/200 mg  4/day  (1 - 1 - 1 - 1) | 812 |
|  | 34 |  |  |  | 1 mg  1/day  (1) | 6 mg  1/day  (1) |  |  |  |  |  |  | 100/25/200 mg  4/day  (1 - 1 - 1 - 1) | 812 |
|  | 29 |  |  |  | 1 mg  1/day  (1) | 6 mg  1/day  (1) |  |  |  |  |  |  | 125/31.25/200 mg  4/day  (1 - 1 - 1 - 1) | 945 |
|  | 27 |  |  |  | 1 mg  1/day  (1) | 6 mg  1/day  (1) |  |  |  |  |  |  | 125/31.25/200 mg  4/day  (1 - 1 - 1 - 1) | 945 |
|  | 25 |  |  |  | 1 mg  1/day  (1) | 6 mg  1/day  (1) |  |  |  |  |  |  | 125/31.25/200 mg  4/day  (1 - 1 - 1 - 1) | 945 |
|  | 22 |  |  |  | 1 mg  1/day  (1) | 6 mg  1/day  (1) |  |  |  |  |  |  | 125/31.25/200 mg  4/day  (1 - 1 - 1 - 1) | 945 |
|  | 8 |  |  |  | 1 mg  1/day  (1) | 6 mg  1/day  (1) |  | 25/100 mg  1/day  (1) |  |  |  |  | 150/37.5/200 mg  4/day  (1 - 1 - 1 - 1) | 1153 |
|  | 6 |  |  |  | 1 mg  1/day  (1) | 6 mg  1/day  (1) |  | 25/100 mg  1/day  (1) |  |  |  |  | 150/37.5/200 mg  4/day  (1 - 1 - 1 - 1) | 1153 |
|  | 1 |  |  |  |  | 6 mg  1/day  (1) |  | 25/100 mg  1/day  (1) |  |  | 50 mg  1/day  (1) |  | 150/37.5/200 mg  4/day  (1 - 1 - 1 - 1) | 1153 |
| (LID) | 0 |  |  |  |  | 6 mg  1/day  (1) |  | 25/100 mg  1/day  (1) |  |  | 50 mg  1/day  (1) |  | 150/37.5/200 mg  4/day  (1 - 1 - 1 - 1) | 1153 |
|  |  |  |  |  |  |  |  |  |  |  |  |  |  |  |
| **014** |  |  |  |  |  |  |  |  |  |  |  |  |  |  |
| (PSG) | 55 | 125 mg  3/day  (1 - 1 - 1) |  |  |  |  |  |  |  |  |  |  |  | 300 |
|  | 53 | 125 mg  3/day  (1 - 1 - 1) |  |  |  |  |  |  |  |  |  |  |  | 300 |
|  | 50 | 125 mg  4/day  (1 - 1 - 1 - 1) |  |  |  |  |  |  |  |  |  |  |  | 400 |
|  | 45 | 125 mg  4/day  (1 - 1 - 1 - 1) |  |  |  |  |  |  |  |  |  |  |  | 400 |
|  | 39 | 125 mg  4/day  (1 - 1 - 1 - 1) |  |  |  |  |  |  |  |  |  |  |  | 400 |
|  | 27 | 125 mg  4/day  (1 - 1 - 1 - 1) |  |  |  |  |  |  |  |  |  |  |  | 400 |
|  | 24 | 125 mg  4/day  (1 - 1 - 1 - 1) |  |  |  |  |  |  |  |  |  |  |  | 400 |
|  | 23 | 125 mg  4/day  (1 - 1 - 1 - 1) |  |  |  |  |  |  |  |  |  |  |  | 400 |
|  | 19 | 125 mg  4/day  (1 - 1 - 1 - 1) |  |  |  |  |  |  |  |  |  |  |  | 400 |
|  | 16 | 125 mg  4/day  (1.5 - 1.5 - 1.5 - 1) |  |  |  |  |  |  |  |  |  |  |  | 550 |
|  | 14 | 125 mg  4/day  (1.5 - 1.5 - 1.5 - 1) |  |  |  |  |  |  |  |  |  |  |  | 550 |
|  | 10 | 125 mg  4/day  (1.5 - 1.5 - 1.5 - 1) |  |  |  |  |  |  |  |  |  |  |  | 550 |
|  | 6 | 125 mg  4/day  (1.5 - 1.5 - 1.5 - 1) |  |  |  |  |  |  |  |  |  |  |  | 550 |
| (LID) | 0 | 125 mg  4/day  (1.5 - 1.5 - 1.5 -1) |  |  |  |  |  |  |  |  |  |  |  | 550 |
|  |  |  |  |  |  |  |  |  |  |  |  |  |  |  |
| **015** |  |  |  |  |  |  |  |  |  |  |  |  |  |  |
| (PSG) | 31 |  | 62.5 mg  1/day  (1) | 250mg  1/day  (1) |  |  |  |  |  |  |  |  | 125/31.25/200 mg  4/day  (1 - 1 - 1 - 1) | 700 |
|  | 24 |  | 62.5 mg  1/day  (1) | 250mg  1/day  (1) |  |  |  |  |  | 1.5 mg  1/day  (1) +  0.75 mg  1/day  (1) |  |  | 125/31.25/200 mg  4/day  (1 - 1 - 1 - 1) | 1090 |
|  | 23 |  | 125 mg  1/day  (1) | 250mg  1/day  (1) |  |  |  |  |  | 1.5 mg  1/day  (1) +  0.75 mg  1/day  (1) |  |  | 125/31.25/200 mg  4/day  (1 - 1 - 1 - 1) | 1140 |
|  | 18 |  | 125 mg  1/day  (1) | 250mg  1/day  (1) |  |  |  |  |  | 1.5 mg  1/day  (1) +  0.75 mg  1/day  (1) |  |  | 125/31.25/200 mg  4/day  (1 - 1 - 1 - 1) | 1140 |
|  | 16 |  | 125 mg  1/day  (1) | 250mg  1/day  (1) |  |  |  |  |  | 1.5 mg  1/day  (1) +  0.75 mg  1/day  (1) |  |  | 125/31.25/200 mg  4/day  (1 - 1 - 1 - 1) | 1140 |
|  | 13 |  | 125 mg  1/day  (1) | 250mg  1/day  (1) |  |  |  |  |  | 1.5 mg  1/day  (1) +  0.75 mg  1/day  (1) |  |  | 125/31.25/200 mg  4/day  (1 - 1 - 1 - 1) | 1140 |
|  | **9** |  | 125 mg  1/day  (1) | 250mg  1/day  (1) |  |  |  |  |  | 1.5 mg  1/day  (1) +  0.75 mg  1/day  (1) |  |  | 150/37.5/200 mg  1/day +  125/31.25/200 mg  3/day  (1 - 1 - 1 - 1) | 1173 |
|  | **4** |  | 125 mg  1/day  (1) | 250mg  1/day  (1) |  |  |  |  |  | 1.5 mg  1/day  (1) +  0.75 mg  1/day  (1) |  |  | 150/37.5/200 mg  1/day +  125/31.25/200 mg  3/day  (1 - 1 - 1 - 1) | 1173 |
|  | **3** |  | 125 mg  1/day  (1) | 250mg  1/day  (1) |  |  |  |  |  | 1.5 mg  1/day  (1) +  0.75 mg  1/day  (1) |  |  | 150/37.5/200 mg  1/day +  125/31.25/200 mg  3/day  (1 - 1 - 1 - 1) | 1173 |
| (LID) | **0** |  | 125 mg  1/day  (1) | 250mg  1/day  (1) |  |  |  |  |  | 1.5 mg 1/day  (1) +  0.75 mg  1/day  (1) |  |  | 150/37.5/200 mg  1/day +  125/31.25/200 mg  3/day  (1 - 1 - 1 - 1) | 1173 |

Suppl. Tab. S1: **Dopaminergic medications.** Type, amount and distribution throughout the day, of dopaminergic medications, and correspondent amount of LEDD, prescribed to each patient at each visit, starting from the time of the PSG recording until the appearance of LID.
